# A Randomized Placebo-controlled Double Blind Clinical Trial of Quercetin for Treatment of Oral Lichen Planus

**DOI:** 10.15171/joddd.2015.005

**Published:** 2015-03-04

**Authors:** Maryam Amirchaghmaghi, Zahra Delavarian, Mehrdad Iranshahi, Mohammad Taghi Shakeri, Pegah Mosannen Mozafari, Amir Hushang Mohammadpour, Fatemeh Farazi, Milad Iranshahy

**Affiliations:** ^1^Associate Professor, Oral and Maxillofacial Diseases Research Center & Department of Oral Medicine, School of Dentistry, Mashhad University of Medical Sciences, Mashhad, Iran; ^2^Associate Professor of Pharmacognosy, Biotechnology Research Center & School of Pharmacy, Mashhad University of Medical Sciences, Mashhad, Iran; ^3^Professor of Biostatistics, Department of Biostatistics & Epidemiology, School of Health, Mashhad University of Medical Science, Mashhad, Iran; ^4^Assistant Professor, Oral and Maxillofacial Diseases Research Center & Department of Oral Medicine, School of Dentistry, Mashhad University of Medical Sciences, Mashhad, Iran; ^5^Associate Professor of Pharmacodynamy & Toxicology, School of Pharmacy & Pharmaceutical Research Center, Mashhad University of Medical Sciences, Mashhad, Iran; ^6^Assistant Professor, Department of Oral Medicine, School of Dentistry, North Khorasan University of Medical Sciences, Bojnurd, Iran; ^7^PhD Student, Department of Pharmacognosy, School of Pharmacy, Mashhad University of Medical Sciences, Mashhad, Iran

**Keywords:** Clinical trial, lichen Planus, quercetin, oral

## Abstract

***Background and aims.*** Standard treatment of oral lichen planus (OLP) includes topical or systemic corticosteroids that have many adverse effects. A trend toward alternative natural or herbal drugs has attended recently. This study was conducted to evaluate the effect of quercetin in treatment of erosive-atrophic OLP.

***Materials and methods.*** Thirty patients participated in this randomized clinical trial from April 2010 to June 2010 (Trial Registration Number: NCT01375101). Patients were randomly allocated in two groups. Both groups received the standard treatment (dexamethasone mouthwash and nystatin suspension). Experimental group received oral 250 mg quercetin hydrate capsules (bid) and the control group received placebo capsules. The pain and severity of the lesions were recorded at the initial visit and the follow-ups. All recorded data were analyzed with chi-square, Mann-Whitney, t-test, Wilcoxon and Friedman tests using SPSS 11.5.

***Results.*** There were no significant differences between the two groups in severity of the lesions and pain in the follow-ups.According to the Friedman test, there was a significant reduction in pain (P = 0.01) and severity indices (P = 0.00) in the case group. These differences were not observed in the control group(P = 0.26,SI; and P = 0.86, PI). No adverse effect of quercetin was reported.

***Conclusion.*** According to the results, no significant therapeutic effect can be considered for quercetin in treatment of OLP.

## Introduction

Oal lichen planus (OLP)is a chronic, inflammatory mucocutaneus disorder with an unknown etiology. Most studies have reported a high incidence of the disease and it is reported that about 0.5-2.5% of the population are affected; the highest incidence of the disease is in the middle aged population with a female predominance.^1,8^Involvement of the oral mucosa is a common finding and can be the only sign of the disease.^2,6,7^ The scalp, nails and genitalia can also be affected. Some studies have suggested malignant potential for OLP.^1,2,6-9^

Although the etiology of OLP is still unclear, there is evidence that it is a complex immunologic disease mediated by cytotoxic cells directed against basilar keratinocytes and resulting in vacuolar degeneration and lyses of basal cells.^1,3,4^In OLP; local inflammatory response is increased because of continued production of cytokines, TNFα, GM-CSF, and IL-6. Alsooxidant/antioxidant balance is altered. Significant increase of lipid peroxidation products and DNA damage has been found within the epidermis.^10^OLP may present as white and red component with the following texture: reticulum, papules, plaque-type, bullous, erythematous, ulcerative. Reticular type needs no treatment and atrophic/erosive type must be treated due to symptoms or malignant potential.^1^

Although corticosteroids are drugs of choice for OLP, attempts have been made to use alternative treatments due to side effects and complications especially in long-term administration. Trends toward natural or herbal origin drugs with antioxidant and anti-inflammatory properties (e.g. quercetin) either individually or in combination with systemic corticosteroids have emerged recently.

Quercetin is an herbal drug which has antioxidant and anti-inflammatory properties. It belongs to flavonoid family and is the most available kind of flavonoids for daily diet. It has the most antioxidant and anti-inflammatory activity among available flavonoids. The anti-inflammatory properties of quercetin have been related to restriction of cytokines including IL12, INFγ, INF α, IL8, cyclooxigenase 2and prostaglandinE.^11,12^ Furthermore, quercetin produces its antioxidant effect by inhibition of free radicals and nitric oxide.^13-15^

Boots et al^16^ found quercetin supplementation improved the antioxidant defence, indicated by the increased total plasma antioxidant capacity. Moreover, quercetin supplementation also reduced markers of oxidative stress and inflammation in the blood of sarcoidosis patients. The effects of quercetin supplementation appeared to be more pronounced when the levels of the oxidative stress and inflammation markers were higher at baseline.^16^

To the best of our knowledge, this is no study on the efficacy of quercetin for lichen planus. Therefore, this study was conducted to evaluate complementary administration of quercetin (in addition to topical corticosteroids) in treatment of erosive- atrophic OLP. This clinical trial was registered in clinicaltrial.gov with the registration number NCT01375101.

## Materials and Methods

### Participants

This prospective randomized double-blind controlled trial was performed in the Department of Oral Medicine at Mashhad University of Medical Sciences, Mashhad, Iran, from April 2010 to June 2010. Thirty five patients were assessed according to eligibility requirements. Five patients were excluded because of their preference to seek treatment in private clinics (N=3) or lack of transportation (N=2). Therefore, 30 patients with clinically and histopathologically detected erosive OLP participated in the clinical trial. Inclusion criteria: 1) Disease duration of more than two month; 2) Absence of dysplasia in histopathologic evaluation; 3) The size of lesion grade ≤2 (Table 1).Study subjects having a history of any type of OLP therapy within the previous month, any mucosal disease or severe systemic disease, pregnancy or breast feeding, lichenoid reaction due to specific etiologies (e.g. drugs or dental restorations), patients using cyclosporine or fluoroquinon because of possible interaction with quercetin or refused to observe the clinician’s advice were excluded from the study.

**Table 1:** Grading of lesion size

Size of lesions	Grading
Normal mucosa=0	Grade 0
0<Size of lesion<1cm	Grade 1
1<Size of lesion<3cm	Grade 2
3cm<Size of lesion	Grade 3

Patients were told they might receive drug or placebo treatment and were educated about the possible side effects of quercetin. This trial was conducted in accordance with ethical principles and was approved by ethics committee of Mashhad University of Medical Science. All the patients signed an informed consent form before the initiation of research. At the patient’s first visit, information including age, gender, disease process, medical history, family history, and clinical signs and symptoms were documented.

### Interventions, Randomization and Blinding

The two study groups were provided with identical capsules (series A and series B). The case group A received capsules containing 250 mg quercetin hydrate (Sigma, St. Louis, US) two times a day and the control group B received capsule containing lactose. Dosage of quercetin was determinate based on other studies, considering half-life of quercatin and consultation with pharmacologist.^16^Determination of whether a patient should be treated by quercetin (group A) or placebo (group B) was made by reference to a statistical series based on a random number. Neither patients nor researchers were aware of which medication was being administered. Only the pharmacist was unblinded but had no contact with study participants. All the patients were instructed to use two capsules daily for 4 weeks. A weekly follow-up was arranged for all patients. Those who did not obtain a complete response from treatment within 4 weeks continued the treatment two times daily and were assessed every week for another four weeks. All the patients received standard treatment for OLP (0.5 mg dexamethasone mouthwash *qid* and 100.000 Unit nystatin suspension *qid*).

### Clinical Assessment

The responses of erosive OLP to quercetin and placebo and dexamethasone were evaluated on the basis of severity index (SI) and pain index (PI) improvement (Tables 2 & 3). All the values for SI and PI were assessed and recorded at the start and the end of each week by two independent researchers who were blinded to the medications for the whole treatment duration and in case of disagreement consensus resolved the issue. A sterile caulis was used to measure the maximum diameter of erosive and atrophic lesions. Grading of size was defined as shown in  Table 1. Severity index was calculated as follows (severity index of several lesion were added together).^17^

**Table 2:** Grading of severity index improvement

Improvement of severity lesion	Grading
N=-0%	Under zero= aggravated lesion
N=0%	0= no improvement of lesion
0<N<25%	1=mild improvement
25%<N<75%	2,3=moderate improvement
75%<N<100%	4= dramatic improvement
N=100%	5=without lesion

**Table 3:** Grading of pain index improvement

Improvement of pain	Grading
N=-0%	Under zero= aggravated pain
N=0%	0= no improvement of pain
0<N<25%	1=mild improvement
25%<N<75%	2,3=moderate improvement
75%<N<100%	4= dramatic improvement
N=100%	5=without pain

Severity index=∑Score of erosive lesion ×grade of size of erosive lesions+∑Score of atrophic lesion×grade of size of atroph

Score of erosive lesion = 2

Score of atrophic lesion = 1.5

Pain or burning sensation was self-assessed by patients using a 10-cm line visual analog scale (VAS). Patients marked the point from 0 (no pain) to 10 (extreme pain) representing their present pain perception. VAS grading was defined as shown in  Table 4.

**Table 4:** Grading of visual analogue (VAS) scores

VAS SCORES	Grading
VAS=0	Grade 0=without pain
0<VAS<3.5	Grade 1= mild pain
3.5<VAS<7	Grade 2= moderate pain
7<VAS<10	Grade 3= severe pain

Improvement of lesions and symptoms were calculated by formulas and based on grading of SI and PI described in Tables 2 & 3. Improvement of severity index =severity index in first visit −severity index in last visit severity index in first visit $\mathrm{Improvement of pain index}=\frac{\mathrm{pain index in first visit}-\mathrm{pain index in last visit}}{\mathrm{pain index in first visit}}$

### Adverse Reactions

In the event of an adverse reaction, it was noted and the patient was put under observation. In case of serious reactions, treatment was discontinued and the subject was sent for treatment to an outside clinic not involved in the research.

### Follow-up Assessment

Patients with complete elimination of the erosion at any time were followed up for one month to detect recurrences. Meanwhile, patients who did not get a complete response continued the treatment 2 times daily and were assessed every week for another 4 weeks. If the SI and PI were0, the treatment was stopped. The patients who still had erosions after 1 month of treatment were advised to continue treatment for another 4 weeks and then were referred for other therapies including topical immunosuppressant, intra-lesional corticosteroids or systemic corticosteroids, or laser therapy.

### Statistical Analysis

Statistical analysis was performed using SPSS 11.0 software for Windows (SPSS Inc, Chicago, USA). The differences in erosive size and VAS scores between the beginning and the end of the treatment in each group were calculated by Wilcoxon signed-rank test and Friedman test. The normality of variables was assessed with Kolmogorov–Smirnov test. The differences in erosive size and VAS scores between the two groups were analyzed by t-test if variables were normal, and by non-parametric Man-Whitney U test if the variables were not normal. All statistical tests were performed at a significance level of P<0.05 (two-tailed).

## Results

Thirty-five patients were initially eligible for the study. Five patients were excluded due to preference to refer to a private center (N=3) or difficulty of transportation (N=2). Thirty patients including 8 men (27%) and 22 women (73%), aged 18 to 72 years, were included in the trial, 15 in the quercetin group and 15 in the placebo group. All patients received dexamethasone mouthwash and nystatin as the standard treatment of OLP. There were no differences between the two groups in age, gender, erosive size, VAS scores, erosive severity, and previous treatment for lichen planus at the start of treatment (P>0.05). The baseline comparison of the two groups is shown in  Table 5.

**Table 5:** Baseline characteristics of the study participants

Characteristics		Quercetin group (N=15)	Placebo group(N=15)	P Value
Age(mean ± SD)		48.26±16.28	44.6±10.22	0.468
Gender, n(%)	Male	2(20%)	5(33%)	0.682
	Female	12(80%)	10(66%)	
Previous treatment, n(%)	No treatment	5(33%)	5(33%)	1.00
n(%)	Treatment	10(66%)	10(66%)	
Pain index (mean±SD)	1.92±0.86	1.80±0.77	0.665
Severity index (mean±SD)	9.40±3.16	9.63±3.83	0.625
Size of lesions (mean±SD)	Erosive	1.46±3.24	2.46±3.97	0.24
(mean±SD)	Atrophic	11.2±8.53	23±40.91	0.74

### Outcome and Estimation

Data from 30 patients, 15 in the quercetin group and 15 in placebo group were analyzed. A significant reduction in erosion severity (P=0.00) and pain (P=0.01) was observed in the quercetin group between follow-ups, but there was no significant reduction in erosive severity index (P=0.26) and VAS scores (P=0.086) in the placebo group. However, there were no significant differences between quercetin and placebo groups in the VAS scores and lesion severity after two, three, and four weeks of treatment (P>0.05).

### Safety Analysis

None of the patients had severe systematic or topical adverse reactions to quercetin during the study.

### Follow-up Analysis

Of all patients who continued treatment until the 4-week assessment, twelve had complete eradication of erosion (SI= 4,5) and seven were in grade4(dramatic improvement). Eleven patients who did not achieve a complete or dramatic improvement response (SI, 1-3) at 4 weeks (8 patients in quercetin group and 3 in the placebo group) continued the treatment for another 4 weeks. One patient who discontinued the initial treatment after completing the entire 8 weeks (severity grade, 1) was referred for other therapy options. Other 10 patients had complete or dramatic improvement at week 12.

VAS scores and erosion severity in quercetin group significantly reduced at weeks 2, 3, 4, and 8. Erosion severity was not significantly different between the two groups at weeks 2, 3, and 8 (Table 6).

**Table 6:** Comparison of sign and symptoms between quercetin and placebo groups before and after 1,2,3,4, and 8 weeks

Severity index	Baseline	Week1	Week2	Week3	Week 4	Week8
Quercetin (N=15)	9.40±3.16	5.93±3.15	4.73±3.23	3.70±3.30	3.23±3.47	2.23±2.93
Placebo (N=15)	9.63±3.83	4.63±2.6	3.70±2.35	2.50±2.45	1.33±1.87	1.10±2.35
Pvalue	0.625	0.231	0.327	0.325	0.126	0.137
Pain index						
Quercetin (N=15)	1.92±0.86	1.07±0.95	0.53±0.66	0.33±0.48	0.23±0.43	0.46±0.51
Placebo (N=15)	1.80±0.77	0.8±0.86	0.86±0.91	0.66±0.89	0.46±0.83	0.53±0.91
Pvalue	0.665	0.427	0.293	0.387	0.618	0.786

## Discussion

OLP is a chronic autoimmune disease and its standard treatment includes topical or systemic corticosteroids. Management of OLP can be challenging as these treatments can have significant side-effects especially when used on a long-term basis or if repeated short courses are needed to control flares. There is a need for safe and effective anti- inflammatory medications to control OLP as a single treatment or in conjunction with corticosteroids.^18^

Quercetin is a potent antioxidant and being used in the management of different systemic conditions such as cancers, hypertension, and cardiovascular diseases, as well as few oral conditions like aphthus ulcers.^19-23^

Quercetin is a flavonoid that possesses a broad spectrum of beneficial properties, including anti-inflammatory and protective effects against oxidative stress, benefits for human endurance exercise capacity, atherosclerosis, thrombosis, hypertension, and arrhythmia as well as modulation of cancer-related multidrug resistance among others, through unknown mechanisms.^15,16,24^The oxidative actions of quercetin dominate the genetically-modified cellular context of malignant cells, thereby promoting apoptosis by modulating the cancerous control of oxidative stress and so it beneficial to premalignant lesions such as OLP.^25^Inflammation is considered to play a pivotal role in OLP pathogenesis by triggering activation of transcription factors such as nuclear factor kappa B (NF-κB), which can be suppressed by quercetin.^26,27^

The purpose of this study was to evaluate the efficacy of quercetin in treatment of erosive OLP. Both case and control groups received topical dexamethasone and nystatin as the standard treatment, and thus the results showed no significant efficacy forquercetin in reducing clinical signs(severity index) and alleviating symptoms(pain and burning sensation) at 1, 2, 3, 4 and 8 weeks. Quercetin was found to be as effective as the placebo in healing erosions at one month. Case group had a significant reduction in SI and PI during 4 weeks whereas in control group these reductions were not significant.

Intragroup analysis showed significant differences in each group but inter-group analysis did not show any significant differences.

Similar studies on the role of quercetin in treatment of OLP have not been published, so a comparison cannot be made at this time. With regards to another oral condition, topical quercetin has been used to treat recurrent aphthus stomatitis and the results indicate that quercetin is effective in complete improvement of lesions in 100% of patients after 10 days.^21^Other natural alternatives such as curcuminoids and aleo vera have been used to treat OLP;^18,28^ however, a comparison is not possible due to differences in research design and drug mechanisms.

Reddy et al^29^evaluated the effectiveness of aloe vera gel in the treatment of oral lichen planus when compared with triamcinolone acetonide and concluded that aloe vera gel can be considered a safe alternative treatment for OLP. Mansurian et al^30^also studied the therapeutic effects of aloe vera mouthwash on OLP and concluded that aloe vera is an effective substitute for 0.1% triamcinolone acetonide.^30^Salazar-Sánchez et al,^28^however, evaluated the efficacy of the topical application of aloe vera in OLP compared with placebo and found no statistically significant differences between groups in relation to pain after 6 and 12 weeks.

Chainani-Wu et al^18^ also found that curcuminoids, at doses of 6000 mg/d in 3 divided doses, are well-tolerated and may prove efficacious in controlling signs and symptoms of OLP.

In addition, Mousavi et al^31^ suggest that Ignatia as a homeopathic remedy has a beneficial effect in treatment of OLP in selected patients.^31^

Sanatkhani et al^32^ evaluated the effect of cedar honey on erosive-atrophic OLP and found it to be effective in the healing of ulcerative lesions with no significant difference compared to the control group.^32^

In the majority of previous studies, the patients in the placebo group did not receive corticosteroids; however, for ethical consideration, topical corticosteroids was administered in the present study. The limited number of patients as well as the short-term treatment and follow-up periods might be responsible for similar treatment efficacy and absence of adverse reactions in the two groups of the present study.

Other factors such as psychological etiologies must be considered in future studies. In one study, Delavarian et al^33^showed that psychological stressors can aggravate OLP and psychological interventions can be significantly effective in treatment of OLP.No significant differences were found in treatment outcomes in the studied groups of the present clinical trial, in which psychological status was not assessed. It seems to the authors that the case group had more stressors (e.g. breast cancer in one patient 6 years before, death of children) and psychological problems (e.g. depression, anxiety) compared with the control group and this could have been responsible for similar treatment outcomes in the two groups.

It is recommended that more research be conducted with larger sample sizes and higher doses of quercetin, probably with longer follow–up periods, and controlling for psychological factors. Topical application of the drug can also be evaluated.

In conclusion, in short-term administration, efficacy and maintenance of efficacy of systemic quercetin were the same as placebo. The drug was safe with no adverse effects.
